# Application of NIR Fluorescent Materials in Imaging and Treatment of Tumors of Different Depths

**DOI:** 10.3390/nano15110811

**Published:** 2025-05-28

**Authors:** Mengdi Yu, Xuan Liu, Shuqiong Wang, Ziyao Qin, Beibei Hu, Zhiwei Li, Shiguo Sun

**Affiliations:** 1 College of Chemical and Pharmaceutical Engineering, Hebei University of Science and Technology, 26 Yuxiang Road, Shijiazhuang 050018, China; 2023105164@stu.hebust.edu.cn (M.Y.); 2201060210@stu.hebust.edu.cn (X.L.); 2023105153@stu.hebust.edu.cn (Z.Q.); 2 Department of Applied Chemistry, Hengshui University, 1088 Heping West Road, Hengshui 053000, China; 15837046193@163.com; 3 Shanxi Key Laboratory of Natural Products & Chemical Biology, College of Chemistry & Pharmacy, Northwest A&F University, Xianyang 712100, China

**Keywords:** near-infrared (NIR), fluorescence imaging (FLI), fluorescence molecular tomography (FMT), tumor, phototherapy, image-guided surgery

## Abstract

Deep-seated tumors present significant diagnostic challenges and pose substantial mortality risks due to their occult anatomical localization. Current diagnostic paradigms predominantly depend on conventional imaging modalities; nevertheless, inherent technical constraints persistently compromise diagnostic precision and therapeutic efficacy. In contrast to traditional methodologies, near-infrared (NIR; 700–1700 nm) fluorescence imaging (FLI) demonstrates superior sensitivity and spatiotemporal resolution, facilitating real-time intraoperative visualization and precision-guided surgical interventions. This paper explores fluorescence materials with tailored structures for tumors at different depths. We critically analyze optimization strategies for NIR fluorescence materials while evaluating their comparative advantages in stratified tissue imaging. This study presents a systematic evaluation of NIR fluorescence molecular tomography (FMT) systems and image reconstruction methodologies. These insights provide feasible ideas for detecting and treating tumors at varying depths in clinical practice. Furthermore, the application of NIR fluorescent materials in tumor diagnosis, navigation-guided surgery, and phototherapy (including photothermal, photodynamic, and immunomodulation therapies) is discussed. Finally, the prospects and challenges of clinical transformation are summarized.

## 1. Introduction

Evolving dietary patterns and lifestyle modifications have driven a global rise in malignant tumor incidence, establishing them as primary mortality determinants impacting global health [1]. Tumor morbidity and mortality persist at elevated levels, necessitating early detection and intervention for effective disease management. Beyond surgical resection, chemotherapy and radiotherapy remain the mainstays in oncology [2,3]. However, therapeutic resistance and severe treatment-related toxicities frequently contribute to tumor relapse and protracted patient morbidity [4].

**Figure 1:**
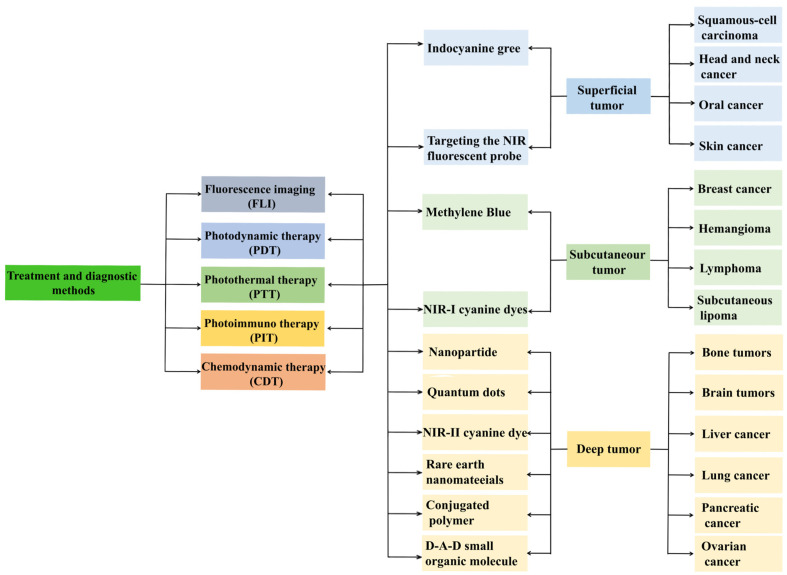
Flowchart of the description of various NIR materials used for specific tumor sites at different depths.

Recent advances demonstrate tumor-targeting fluorophores as clinically translatable agents for imaging-guided oncotherapy. Developed NIR probes span methylene blue (MB), carbon nanotube (SWCNTs) [5], inorganic nanoparticles [6,7], quantum dots (QDs) [8,9,10], rare-earth nanomaterials [11,12], organic small molecules [13], fluorescent dyes [14], and conjugated polymers [15]. FLI modalities are categorized into visible VIS (400–700 nm), NIR-I (700–900 nm), and NIR-II (1000–1700 nm) regimes by emission spectra. In vitro murine models demonstrated that deep-tissue imaging is compromised by intrinsic autofluorescence, while NIR wavelengths exhibit significantly lower scattering coefficients compared to VIS [16]. As shown in Figure 1, we integrated NIR-responsive materials with combinatorial therapeutic strategies, including chemotherapy, photodynamic therapy (PDT), photothermal therapy (PTT), photoimmunotherapy (PIT), photoacoustic therapy (PA), chemodynamic therapy (CDT), and multimodal regimens, and achieved enhanced spatiotemporal precision in tumor targeting and treatment efficacy [17].

In systematically reviewing advancements in NIR-II fluorescent probes for tumor surgery navigation, we identified critical limitations in existing review articles, notably their narrow analytical dimensions and restricted application scopes. For instance, Zia Ullah et al. comprehensively characterized NIR-II probes (e.g., QDs and organic small molecules) from a materials chemistry perspective, yet failed to establish comparative frameworks between NIR-II and NIR-I probes or to address anatomical tumor localization in probe selection [18]. Similarly, while Homan Kang’s team innovatively integrated cancer immunotherapy with NIR FLI, they omitted synergistic therapeutic modalities like PTT and PDT [19].

This research establishes a clinically stratified anatomical framework that correlates probe emission spectra (700–1700 nm) with malignant tissue penetration depth and spatial resolution across anatomical hierarchies. Employing an evidence-based analytical framework, we methodically evaluate NIR-guided surgical interventions integrated with combinatorial phototherapies (PTT/PDT/PIT), while synthesizing NIR fluorescence molecular tomography (FMT) architectures and computational reconstruction paradigms, bridging critical gaps in systematic investigations. Table 1 summarizes the applications of NIR fluorescent materials with different structures in tumor imaging and therapy at varying depths.

## 2. Superficial Tumor

Superficial tumors comprise epithelial-origin malignancies primarily localized to cutaneous/submucosal layers, including squamous cell carcinoma, head-neck cancers, oral epidermoid carcinoma, and melanoma [40]. While early intervention mitigates metastatic dissemination, therapeutic management persists as a clinical challenge owing to surgical morbidity and chemotoxicity [41]. Current diagnostic armamentarium integrates computed tomography (CT), ultrasound (US), magnetic resonance imaging (MRI) [42,43], positron emission tomography (PET) [44], and single-photon emission computed tomography (SPECT). FLI surpasses conventional modalities through real-time capability, non-invasiveness, and superior target-to-background ratios, positioning it as a transformative diagnostic alternative [45]. Clinically validated NIR probes like indocyanine green (ICG) dominate superficial tumor imaging, while targeted NIR fluorophores exhibit enhanced tumor-specific accumulation.

### 2.1. Indocyanine Green (ICG) Fluorescent Dyes

ICG, an amphiphilic water-soluble fluorophore, exhibits excitation at 778–806 nm with 835 nm peak emission in biological matrices [46]. It is the sole U.S. Food and Drug Administration (FDA) approved NIR contrast agent for surgical navigation [47]. This photosensitizer generates reactive oxygen species (ROS) under NIR irradiation, inducing cytotoxic effects against malignant and microbial targets [48]. Its clinical utility stems from its negligible systemic toxicity [49]. Integrated with NIR imaging, ICG enables early superficial tumor detection, sentinel node mapping, cervical lymphadenectomy, and chemotherapy targeting within the NIR-I window [50]. Wang et al. implemented ICG-guided NIR laparoscopy for precise esophagectomy with esophagogastric reconstruction [51]. This technique achieved intraoperative lesion demarcation, surgical margin preservation, lymphatic mapping, and anastomotic perfusion assessment. Although ICG has been approved by the FDA as a fluorescent imaging agent, certain inherent limitations persist that must be systematically addressed to optimize its clinical utility and advance surgical outcomes. For instance: (1) poor light stability, (2) rapid systemic clearance, (3) tumor-targeting deficiency [52], and (4) aqueous-phase aggregation causing quantum yield attenuation [53].

ICG-based FGS exhibits inherent limitations. To augment photostability, structural modifications of ICG via the incorporation of rigid moieties or electron donor-acceptor (D-A) substituents enable precise modulation of its excited-state energy distribution. This molecular engineering strategy suppresses non-radiative relaxation pathways and mitigates photobleaching by stabilizing the fluorophore’s electronically excited states, thereby preserving fluorescence intensity under prolonged illumination. Owing to self-quenching, ICG in solution displays reduced fluorescence quantum yield and rapid hepatic clearance post-intravenous injection [54]. Encapsulating ICG within nanoparticles addresses these constraints by improving stability, prolonging circulation, and enhancing tumor-specific accumulation [55].

To enhance targeting precision, covalent conjugation of ICG with molecular ligands (e.g., antibodies, peptides) enables the construction of ligand-functionalized ICG complexes. Ito et al. demonstrated ICG-conjugated anti-Podoplanin probes for NIR visualization of OSCC xenografts in murine models [22]. Albumin-conjugated ICG further demonstrates enhanced hydrolysis resistance and a 16.8% increase in photoluminescent quantum yield relative to unbound ICG [56]. This strategy exploits ligand-receptor molecular recognition mechanisms to achieve active accumulation in pathological regions, thereby amplifying signal-to-background ratios during fluorescence-guided interventions.

**Figure 2:**
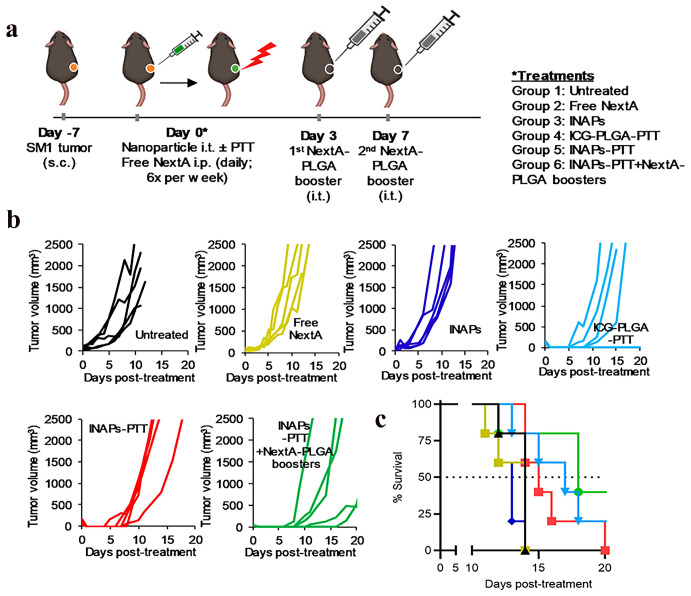
Photothermal nanotherapy (INAP-PTT) inhibits melanoma progression and extends survival in B16-F10 murine models. (**a**) Therapeutic workflow for precision thermotherapy. (**b**) Longitudinal tumor volumetrics (*n* = 5) and (**c**) Kaplan–Meier analysis reveal extended recurrence-free survival with adjuvant epigenetic modulation (INAP-PTT + NextA-PLGA). Adapted with permission from references [51]. Copyright 2020, MDPI.

The encapsulation of ICG within inorganic or organic nanocarriers (e.g., silica nanoparticles, liposomes, or polymeric micelles) leverages the light-scattering or energy-absorption properties of the carrier matrix to mitigate photodegradation kinetics. This strategy not only prolongs the photostability of the fluorophore but also enhances photothermal conversion efficiency, thereby accelerating therapeutic efficacy in tumor ablation. For instance, Ledezma-DK et al. developed a nanoemulsion synthesis protocol to co-encapsulate ICG and Nexturastat A (NextA) within monodisperse poly (lactic-co-glycolic) acid (PLGA)-based nanoparticles (ICG-NextA-PLGA; INAPs) (Figure 2a). Therapeutic cohorts receiving epigenetic monotherapy exhibited tumor growth curves comparable to untreated tumor-bearing mice (Figure 2b). Compared with untreated controls, near-daily systemic NextA administration conferred no tumor progression benefit. In contrast, the ICG-PLGA-PTT group and the combinatorial INAPs-PTT + NextA-PLGA regimen demonstrated significantly attenuated tumor progression. Kaplan–Meier survival analysis revealed median survival durations of 18 days (INAPs-PTT + NextA-PLGA), 17 days (ICG-PLGA-PTT), and 14 days (untreated controls) (Figure 2c). These findings establish INAP as an epigenetic regulator whose combinatorial delivery platform delays tumorigenesis and extends median survival [51].

### 2.2. Targeting the NIR Fluorescent Probe

**Figure 3:**
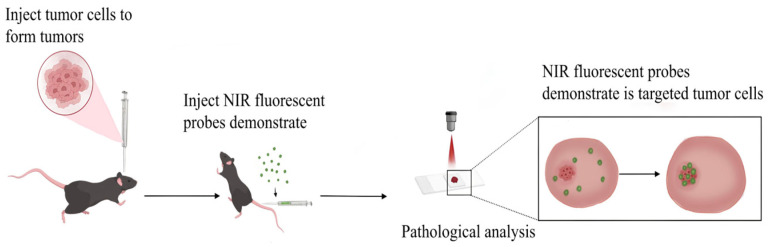
Schematic diagram of NIR fluorescent probe specifically targeting tumors.

Owing to enhanced tumor target specificity, active targeting strategies and activatable probes present novel opportunities for intraoperative navigation. NIR-II fluorescent probes demonstrate superior efficacy in tumor imaging owing to their target selectivity, high spatial resolution, and deep-tissue penetration capabilities [57], as shown in Figure 3.

ICG can be conjugated with functionalized nanoparticles through immobilization, doping, or covalent coupling strategies, enabling ligand-mediated tumor targeting [58]. Von Kiedrowski et al. [59] engineered an NIR fluorescent cyclic Alpha-melanocyte-stimulating hormone (α-MSH) peptide conjugate incorporating indocyanine dye, demonstrating melanocortin 1 receptor (MC1R)-specific binding capacity for precision diagnosis of malignant melanoma. The NIR-II fluorophore Nd-Mn-MMP1Ab luminescent probe exhibited selective binding to Human Tongue Squamous Cell Carcinoma (Cal-27) cells, enabling precise OSCC tracking through targeted molecular recognition [60]. Notably, TM1-IR680 conjugated with gastrin-releasing peptide receptor (GRPR)-targeting peptides enabled intraoperative visualization of tumor margins and lymphatic metastases in orthotopic oral cancer murine models [61]. Furthermore, MC1R-directed fluorescent nanoprobes (MSH-TPE-BBT NPs) displayed enhanced tumor permeability, photophysical stability, biocompatibility, and specific accumulation in human/murine melanoma xenografts [62].

## 3. Subcutaneous Tumor

Multiple fluorophores have been developed for subcutaneous tumor imaging, yet VIS/NIR-I-based approaches remain suboptimal due to shallow tissue penetration and intense autofluorescence. Select NIR-I probes (such as MB, cyanine derivatives, ICG) achieve emission tailing beyond 1000 nm into the NIR-II regime, enabling enhanced imaging contrast and therapeutic efficacy in subcutaneous malignancies.

### 3.1. Methylene Blue (MB)

MB, a fluorescent dye first identified in 1876, has been applied across diverse scientific disciplines. Recently, MB has been utilized in intraoperative FLI, emitting NIR fluorescence at 700 nm. This dye is cost-effective, readily available, and demonstrates low systemic toxicity, achieving a maximal tissue penetration depth of 4 mm [63,64]. Current applications include intraoperative ureter visualization, parathyroid gland localization, pancreatic tumor delineation, breast cancer margin assessment, and sentinel lymph node mapping [65]. However, its limited diagnostic efficacy and shallow tissue penetration restrict its utility in subcutaneous tumor detection.

MB exhibits pronounced absorption and fluorescence emission within both the VIS NIR-I, enabling dual-modality visualization through FLI and photoacoustic imaging. MB demonstrates preferential biodistribution in endocrine tissues, serving as a surgical adjunct for thyroid/parathyroid procedures through high-dose intravenous administration (3–7.5 mg/kg), enabling visual identification of hyperplastic glands via chromatic differentiation [66]. Breast cancer exhibits metastatic tropism for pulmonary and cerebral tissues [67], and evidence confirms mortality reduction through early intervention strategies [68]. The high glandular density of breast parenchyma compromises mammographic sensitivity [69]. Intravenous MB administration enables NIR fluorescence-based tumor detection, with a radical mastectomy cohort (*n* = 12) achieving measurable fluorescence signals at 1 mg/kg dosing (mean TBR = 1.578 ± 0.36) [70]. Notably, Wu et al. [64] established MB’s efficacy in rodent sentinel lymph node mapping, demonstrating superior lymphatic mapping capability versus ICG.

### 3.2. NIR-I Cyanine Dyes

Cyanine-derived organic fluorophores exhibit elevated quantum yields, strong molar absorptivity, facile synthesis, and biocompatibility [71,72]. However, conventional NIR-I cyanine dyes (700–900 nm) exhibit inherent limitations in clinical oncology, primarily restricted to subcutaneous tumor applications due to their sub-optimal tissue penetration depth (<5 mm) and susceptibility to photon scattering/autofluorescence interference in deeper anatomical regions. These constraints arise from the rapid attenuation of NIR-I wavelengths in biological tissues and spectral overlap with endogenous chromophores (e.g., hemoglobin, melanin), which collectively compromise imaging contrast and therapeutic precision for visceral or orthotopic malignancies.

Breast cancer is the most common tumor. Early-stage intervention through screening and targeted therapies significantly improves survival outcomes [68,73]. To address ICG’s limited tissue penetration from short-wavelength emission, π-extended ICG derivatives conjugated with Human Epidermal Growth Factor Receptor 2 (HER2+)/Epidermal Growth Factor Receptor (EGFR+) antibodies enable deep-tumor NIR imaging in HER2+/EGFR+ subtypes [74]. The ERα-targeted probe IRDye800CW-E2, which combines the cyanine fluorophore IRDye800CW with ethinylestradiol (ERα ligand), demonstrated rapid tumor accumulation (<4 h post-injection) with sustained tumor-to-background contrast (TBR 4–48 h) in murine models, validating its clinical potential for early detection [24]. Preclinical studies confirm HER2+ Affibody-IR700 conjugates mediate targeted ablation of HER2+ tumors in xenograft models, achieving 72% volume reduction within 24 h post-PIT [75].

**Figure 4:**
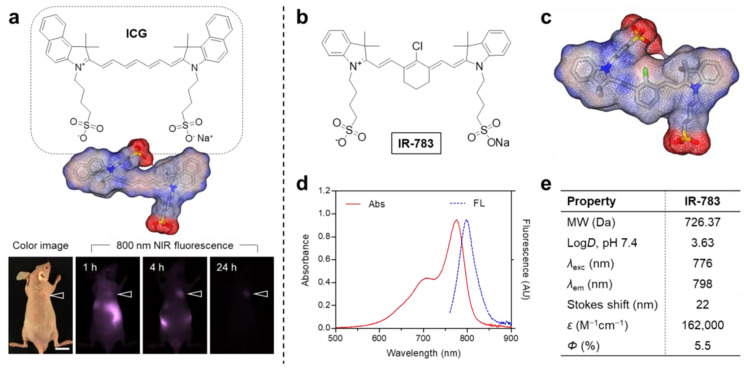
(**a**) ICG chemical structure and in vivo tumor targeting (arrowheads indicate tumor sites; scale bar = 1 cm). (**b**) IR-783 chemical structure and (**c**) 3D electrostatic potential map (red: negative; blue: positive; gray: hydrophobic regions). (**d**) IR-783 absorption/emission spectra in PBS (pH 7.4). (**e**) Computed physicochemical properties of IR-783, including log*D* at pH 7.4 and surface charge distribution (calculated using Marvin/JChem plugins, ChemAxon, Budapest, Hungary). Reproduced with permission from Ref. [76]. Copyright 2024, MDPI.

IR-783, a commercial-grade NIR heptamethine dye, has been investigated in breast and prostate cancer research. Distinct from other commercial heptamethine cyanine dyes, IR-783 demonstrates unique physicochemical properties (Figure 4) [76]. Although structurally analogous to ICG, IR-783 shows minimal tumor uptake within 24 h post-injection (Figure 4a). Its heptamethine skeleton contains a chlorocyclohexenyl ring and dual sulfonate side chains (Figure 4b), improving aqueous solubility. A key structural divergence from ICG is the racemic chlorine atom on the heptamethine cyclohexene ring, which facilitates tumor-selective accumulation via albumin adduct formation. Owing to its hydrophilicity, IR-783 absorption and fluorescence spectra were characterized in PBS (pH 7.4) (Figure 4d), revealing a 776 nm absorption peak, 798 nm emission maximum, and 22 nm Stokes shift (Figure 4e). IR-783 also displays a moderate molar extinction coefficient (ε = 162,000 mol^−1^·cm^−1^) and quantum yield (Φ = 5.5%). These results confirm that IR-783 has great potential in the treatment of tumor cells.

## 4. Deep Tumor

Visceral tumors located in deep anatomical regions pose diagnostic challenges with high mortality upon clinical presentation, encompassing intracranial neoplasms, osseous malignancies, and visceral carcinomas (prostate, hepatic, pulmonary, etc.). Conventional VIS/NIR-I FLI suffers from tissue scattering and autofluorescence-induced compromised resolution and elevated false-positives [16]. Recent developments in NIR-II fluorescent materials have encompassed nanoparticles, QDs, cyanine dyes, rare-earth nanomaterials, conjugated polymers, and donor-acceptor-donor (D-A-D) organic small molecules, overcoming these aforementioned limitations. NIR-II FLI attenuates scattering effects, achieves ~10 mm tissue penetration, and suppresses autofluorescence, enabling precise tumor delineation [77]. This breakthrough facilitates deep-tissue visualization [78] and emerges as a transformative preclinical tool [79]. Its dual functionality spans contrast-enhanced cerebrovascular/cardiac imaging [80,81] to mechanistic investigations in neurodegeneration [82], bone repair [83], gastrointestinal diseases [84,85], kidney diseases [86], cervical diseases [87], diabetic angiopathies [88], and contrast-guided theranostic platforms. Despite their potential, all NIR-II fluorescent materials face inherent trade-offs. To objectively evaluate their clinical applicability, Table 2 provides a comparative analysis of their advantages (e.g., penetration depth, SNR) and limitations (e.g., toxicity, synthesis complexity) in imaging tumors located at deep tissue sites.

### 4.1. Nanoparticle

Fluorescent nanoparticles demonstrate robust luminescence and stability, serving as effective cellular fluorophores. Surpassing conventional modalities, these nanoparticles provide enhanced biosafety through non-radioactive, non-invasive operation, establishing radiation-free diagnostic paradigms [102]. Their tumor imaging merits include: (1) Superior biocompatibility, (2) Extended circulatory half-lives, (3) Passive targeting via EPR-mediated tumor accumulation, (4) High payload capacity for hydrophobic therapeutics, and (5) Facile surface functionalization enabling receptor-specific targeting [89]. However, persistent challenges remain in probe design, stability, and clinical applicability, necessitating further breakthroughs to address critical limitations.

NIR-II nanoparticles enable synergistic integration of PTT, PDT, and CDT through wavelength-specific photoconversion, ROS generation, and Fenton/Fenton-like catalytic cascades. NIR-II-responsive Fe-doped carbon nanoparticles (FDCNs) were engineered via a one-pot hydrothermal method for combined PTT and CDT. Released iron ions catalyze H_2_O_2_-to-hydroxyl radical (OH) conversion, enabling CDT, while achieving a 36.3% photothermal efficiency [103]. Hydrophilic polymer-modified two perylene monoxide and one diaminoanthraquinone (2PMI-AQ) self-assembled into nanoparticles, synergizing PTT and PDT [104]. PDT clinically targets endoscopically accessible malignancies, including bladder and lung cancers [105]. Zhang X et al. [106] developed NIR-II-targeted nanoparticles (NPER/BO-PDT), which triggered immunogenic cell death, adaptive immunity, and tumor suppression under NIR irradiation, demonstrating effective PDT. PTT, a non-invasive modality, shows promise for tumor ablation with minimal invasiveness [107]. NIR-II-driven semiconductor polymer nanoparticles (SPNs) [108] enabled PA-guided U87 glioma PTT. Li et al. [109] engineered AE105 peptide-functionalized CH4T@MOF-PEG-AE nanoprobes, peaking in fluorescence intensity (TBR = 4.0) at 12 h post-injection with 30.4% photothermal efficiency. MR/NIR-II-guided PTT achieved complete glioma ablation.

**Figure 5:**
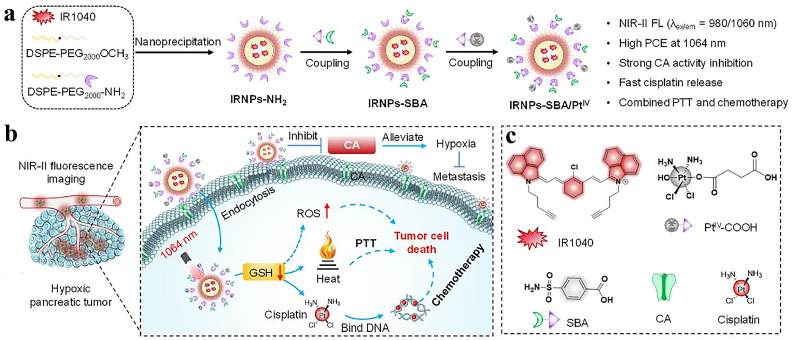
(**a**) Synthesis scheme of IRNPs-SBA/PtIV. (**b**) Working mechanism of IRNPs-SBA/PtIV for NIR-II fluorescence-guided PTT/chemotherapy in pancreatic tumors (1064 nm excitation). (**c**) Molecular structures of IR1040, PtIV–COOH, SBA, cisplatin, and CA representation. Adapted with permission from Ref. [7]. Copyright 2024, Elsevier.

Pancreatic malignancy, characterized by rapid metastasis and elusive early-stage detection, poses significant clinical challenges. The immunosuppressive tumor microenvironment renders suboptimal therapeutic outcomes for conventional radio-chemotherapeutic regimens and immunotherapeutic approaches. NIR-II phototheranostics demonstrates unique advantages in pancreatic tumor resection and ablation [6]. Figure 5a illustrates the synthesis process of NIR-II fluorophore (IRNPs-SBA/PtIV), a cisplatin-prodrug nanoparticle exhibiting intense fluorescence emission at 980 nm excitation. Under 1064 nm irradiation, this system synergizes PTT and chemotherapy to suppress pancreatic tumor progression (Figure 5b) [7]. Engineered NIR-IIb AIE nanoparticles achieve 1550 nm-mediated high-precision glioblastoma delineation at 5.9 mm tissue depths [110]. Clinical trials now explore image-guided phototherapeutic modalities for enhanced precision and safety in neuro-oncological interventions.

### 4.2. Quantum Dots (QDs)

QDs are considered ideal FGS nanoprobes due to their distinct optical properties and superior fluorescence performance, enabling high-fidelity tumor imaging. Nevertheless, intrinsic limitations of NIR-II QDs, which include inherent toxicity, suboptimal targeting efficiency, and limited solubility [91], pose critical barriers to advancing in vivo imaging contrast and spatiotemporal resolution, substantially constraining their clinical translation in FGS [111].

Intrinsic limitations of Ag_2_S QDs, which include suboptimal targeting and aqueous instability, demand structural optimization for theranostic implementation. Zebibula et al. engineered hydrophobic NIR-II PbS@CdS QDs via silica/amphiphilic polymer (Pluronic F-127) bilayer encapsulation [112]. The PbS@CdS@SiO_2_@F-127 nanocomposites demonstrated aqueous dispersibility (Φ = 5.79%) and enabled 950 µm-depth cerebral FLI in murine models. RCA-synthesized Programmed cell Death 1-ligand 1 (PD-L1) aptamer/C-rich DNA scaffolds templated Ag^+^-chelating pApt-Ag_2_S QDs for PD-L1 tumor targeting [91]. Ligand-exchange/amide-condensation strategies yielded tumor-targeting Ag_2_S@PEG-ABS probes functionalized with 4-(2-aminoethyl)benzenesulfonamide, achieving precision FLI. PEGylation conferred enhanced hydrosolubility and photothermal stability (η = 45.17%), validating Ag_2_S@PEG-ABS as a potent colorectal cancer theranostic agent [38].

Intracranial neoplasms are histologically heterogeneous with infiltrative growth patterns, rendering complete resection technically challenging under visual guidance [94]. To overcome these constraints, Angiopep-2-functionalized Ag_2_S QDs were synthesized via carbodiimide-mediated conjugation, demonstrating biocompatibility at concentrations <100 μg/mL. In vivo studies using subcutaneous xenografts revealed preferential accumulation of Angiopep-2-QDs, suggesting active glioma-targeting properties [113]. FGS further provides multimodal surgical guidance for colorectal malignancies, integrating phototheranostic applications (PTT/PDT/PIT) with intraoperative navigation [114]. These findings collectively validate QDs as promising theranostic platforms for deep-tissue oncological imaging.

### 4.3. NIR-II Cyanine Dye

NIR-II cyanine dyes demonstrate advantageous properties such as low cytotoxicity, superior biocompatibility, negligible autofluorescence, and diminished background signals. Clinically relevant fluorophores, IR-1048, IR-1061, and IRDye800, have been utilized for preclinical imaging of deep-seated tumors [112]. Nevertheless, commercial NIR-II cyanine dyes are constrained by inherent limitations: (1) inadequate target specificity, (2) poor aqueous solubility, and (3) low photothermal efficiency [79,94].

**Figure 6:**
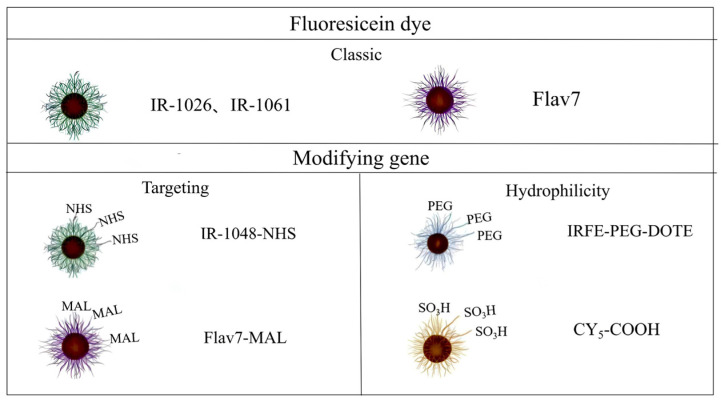
Classification of NIR-II cyanine dyes modified by conventional and modifying groups.

Cyanine derivatives were functionalized with reactive moieties such as N-hydroxysuccinimide (NHS) and maleimide (MAL) groups to enable covalent conjugation with targeting antibodies or peptides, thereby enhancing probe specificity (Figure 6). Lu et al. developed NIR-II fluorophore (H10@FSH), an NIR-II fluorophore demonstrating ovarian-specific targeting and dual-mode PA/FGS navigation for ovarian cancer lesions, enhancing patient theranostic efficacy [115]. Zhang et al. [116] developed a GRPR-targeted probe by conjugating RM26 with IRDye800CW, enabling NIR-II image-guided resection of malignant brain tumors. Novel bone-targeted fluorescent probes improve spatial resolution and specificity in noninvasive metastasis imaging. Sun et al. [31] engineered an NIR-II probe using IRDye800CW-NHS and trastuzumab, demonstrating that BTZ/Fe^2+^@BTF/ALD suppresses bone tumor progression via synergistic NIR-II PTT, chemotherapy, and CDT in a 4T1 murine model. Cy5.5-alendronate conjugates specifically target osteoblastic prostate cancer via NIR fluorescence [117].

Strategic incorporation of poly(ethylene glycol) (PEG) chains or sulfonic acid (-SO_3_H) groups further optimized aqueous solubility and biocompatibility profiles. The inherent hydrophobicity of squaraine (SQ) dyes was addressed through functionalization of each fluorene unit with azide-terminated alkyl side chains, enabling conjugation with poly(oligoethylene glycol methyl ether methacrylate) (POEGMA) to yield water-soluble SQ-POEGMA conjugates with enhanced biocompatibility and photothermal efficacy [118]. Concurrently, Hongyun Qian et al. [119] developed hydrophilic quaternary sulfonate cyanine (HQS-Cy) dyes, where strategic incorporation of sulfonic acid groups and amphiphilic polypeptide encapsulation synergistically improved aqueous solubility while maintaining favorable biocompatibility profiles.

### 4.4. Rare Earth Nanomaterials

Rare-earth-doped nanoparticles have emerged as one of the most promising candidates for NIR-II bioimaging owing to their unique optical properties in this spectral region, including narrowband emission [120], exceptional photostability, deep-tissue penetration, prolonged luminescence lifetimes, and resistance to photobleaching [95,96]. However, the performance of rare-earth-based nanomaterials in NIR-II imaging exhibits significant divergence due to variations in dopant selection and structural engineering. Several limitations persist in current systems: (1) complex synthesis processes for multilayered core-shell architectures (e.g., NaYF4:Yb/Tm@NaYF4:Nd) requiring specialized techniques like thermal decomposition or co-assembly, posing scalability challenges [121]; (2) fluorescence quenching leading to diminished quantum yields; (3) unresolved biosafety concerns regarding long-term biocompatibility; and (4) intrinsic hydrophobicity necessitating surface modifications.

To address the limitations of rare-earth nanomaterials, strategic approaches such as dopant integration, surface functionalization, and protective shell encapsulation can be implemented. In Nd-Yb-Er co-doped NIR-II systems, multilayer core-shell architectures effectively suppress luminescence quenching [122]. Certain rare-earth ions demonstrate pronounced cytotoxicity, necessitating encapsulation within protective shells or coatings prior to biological applications to ensure biocompatibility. Notably, Yana Liu et al. developed a multifunctional platform (UCNPs@MIL-PEG) by coating PEG-functionalized metal-organic frameworks (MOFs) onto upconversion nanoparticles (UCNPs), which exhibited high biocompatibility in cytotoxicity assays for cancer theranostics [123]. To enhance aqueous dispersibility, surface modifiers such as PEG and Tween 20 (Tw) are typically grafted onto rare-earth nanoparticles (RENPs) [124].

Rare-earth luminescent probes exhibit versatile optical properties, encompassing both upconversion and downconversion emissions [125]. Energy upconversion, a nonlinear optical process, involves sequential absorption of multiple low-energy photons followed by intermediate energy-level transitions to emit higher-energy photons. This phenomenon primarily originates from the distinct electronic configurations and energy-transfer dynamics of lanthanide ions (e.g., Er^3+^, Tm^3+^, Ho^3+^, Pr^3+^, and Nd^3+^). The efficiency is governed by synergistic interactions between sensitizers and activators. Lanthanide-based nanoprobes represent an emerging class of NIR-II nanomaterials gaining prominence in bioimaging applications. As a primary dopant, Nd^3+^ exhibits optimal performance at 1–5% molar ratios, beyond which concentration quenching diminishes photoluminescence efficiency [126]. Precise tuning of Yb^3+^ and Er^3+^ content enhances local surface plasmon resonance (LSPR) absorption in low-aspect-ratio gold nanorods under 980 nm excitation, boosting PTT efficacy. Precise tuning of Yb^3+^ and Er^3+^ content enhances LSPR absorption in low-aspect-ratio gold nanorods under 980 nm excitation, boosting PTT efficacy [127]. Thus, rare-earth composition directly modulates emission wavelength and intensity. For deep-tumor imaging, surface modification with BSA, PEG, HA, or PLA improves blood circulation half-life, minimizes macrophage clearance, enhances tumor accumulation, and optimizes biocompatibility, solubility, and targeting [128]. Spatial separation of Nd^3+^ (sensitizer) and Er^3+^ (activator) across distinct layers maximizes 800 nm absorption while minimizing rare-earth cross-relaxation [129]. Yb/Ho/F-co-doped TiO_2_ generates ROS under 808 nm irradiation while exhibiting intensified upconversion fluorescence for deep-tissue imaging [130].

Skeletal metastases predominantly manifest in breast, prostate, and lung carcinomas via hematogenous dissemination [131]. Osteotropic malignancies present complex pathogenesis characterized by occult progression, elevated morbidity/mortality, and high recurrence rates, severely compromising terminal-stage patient prognosis [132]. He et al. engineered a ligand-free NIR-II fluorophore (RENPs@DSPE-mPEG) with intrinsic bone affinity, establishing a radiation-free diagnostic modality for skeletal mapping [133]. Antibody-functionalized rare-earth composites enable multimodal imaging (VIS/NIR-II down-/up-conversion) for precise lung adenocarcinoma delineation and intraoperative guidance [134]. Chen et al. developed NIR-II fluorophore (UiO-66-NH2@AuNS) core-shell nanostructures with NIR-II photothermal ablation capacity, where localized hyperthermia enhanced tumor perfusion while ameliorating hypoxic microenvironments [135].

### 4.5. Conjugated Polymer

Conjugated polymers have gained widespread application in NIR-II imaging owing to their advantageous properties, including high extinction coefficients, superior photothermal conversion efficiency, broad emission spectra, and facile synthesis [136,137]. Nevertheless, engineering conjugated polymers that simultaneously demonstrate optimal NIR-II fluorescence and PTT performance faces three key challenges: (1) limited aqueous solubility, (2) pronounced non-radiative decay causing substantial aggregation-caused quenching [138], and (3) inherently low NIR-II quantum yields in most developed systems, consistent with the energy gap law [98].

The above defects can be improved by optimizing conjugated polymer probes through post-polymerization modification or direct copolymerization to introduce polar/ionic groups and construct core-shell nanostructures. Structural integration of non-conjugated spacers yielded water-soluble nanoparticle (BTC12 NPs), enabling multimodal NIR-II FLI/PA/PTT imaging with 46.8% photothermal efficiency for tumor demarcation. To circumvent excessive non-radiative decay-induced fluorescence quenching, AIE-active NIR-II polymers were synthesized via Stille polycondensation. Dual structural engineering (AIE unit optimization and non-conjugated backbone incorporation) endowed BCT1 with αAIE = 3.27, 70.51% photothermal efficiency, and intense NIR-II emission [138]. Electron-donating moieties (thiophene derivatives) enhance conjugated polymer quantum yields [139]. Alkyl-functionalized bithiophene donors enabled the water-soluble nanoparticle (TTQ-2TC NPs) synthesis with organic solubility and bright NIR-II fluorescence [140]. Electron-acceptor density modulation through tetra-thiophene cores and alkyl side-chain engineering boosted radiative rates and NIR-II brightness [141].

**Figure 7:**
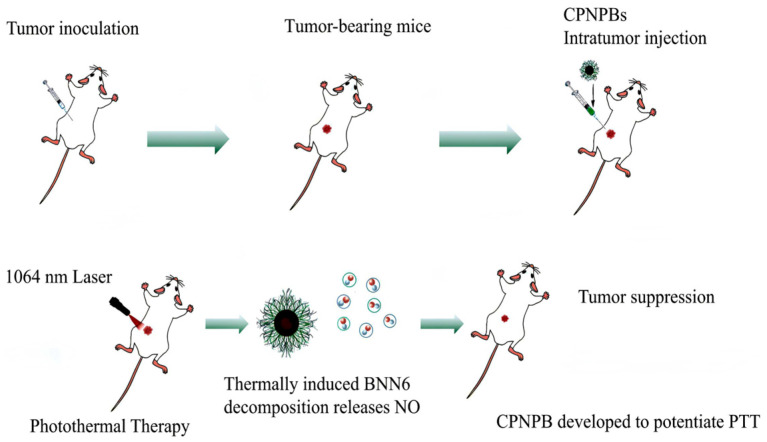
The therapeutic effect of NIR-II conjugated polymer CPNPBs on PTT in tumor mice.

A NIR-II-responsive conjugated polymer nanotheranostic platform (CPNPB) incorporating a nitric oxide (NO) donor has been developed to potentiate PTT (Figure 7). The system comprises three functional components: (1) a conjugated polymer backbone (IN-NDI), (2) a NO-releasing moiety (BNN6), and (3) an amphiphilic stabilizer (F127). This architecture demonstrates remarkable biocompatibility. The platform exhibits broad NIR-II absorption with 55.6% photothermal conversion efficiency, facilitating efficient heat generation at 1064 nm. Thermally induced BNN6 decomposition releases NO, synergistically enhancing the PTT efficacy [142].

### 4.6. D-A-D Small Organic Molecule

D-A-D-type organic small molecules exhibit distinct advantages: well-defined structures, tunable optoelectronic properties, favorable metabolic profiles, substantial Stokes shifts, remarkable stability, and excellent biocompatibility. These characteristics render them particularly attractive for developing organic NIR-II fluorescent probes [100,101]. Nevertheless, fundamental limitations, including aggregation-caused quenching and the energy gap law, constrain their NIR-II quantum yields [143].

A D-π-A-π-D structured fluorophore (CED) was engineered into water-dispersible nanoparticles via DSPE-mPEG encapsulation, yielding a bright NIR-II emitter [144]. The D-A-D framework incorporated diketopyrrolopyrrole (DPP) as a strong acceptor to promote electron delocalization, complemented by cyclopentadithiophene (CPDT) as a secondary acceptor. The extended π-conjugation and intermolecular interactions risked aggregation-caused quenching. Isooctane side chains were grafted onto CPDT to suppress intermolecular interactions and prevent quenching. 3,4-Ethylenedioxythiophene (EDOT) π-bridges were introduced between CPDT and DPP to enhance quantum yield. This design extended conjugation, improved charge transfer, and induced molecular distortion. These fluorophores demonstrate intense absorption and bright NIR-II emission, permitting accurate tumor delineation and localized PTT [145].

**Figure 8:**
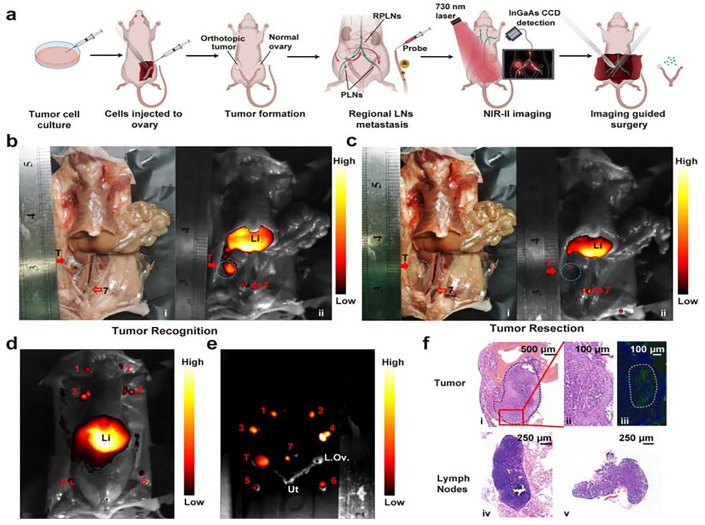
NIR-II fluorescence imaging of orthotopic microtumors and lymph nodes. (**a**) S Experimental design for ovarian cancer modeling and imaging. (**b**,**c**) Surgical field visualization: (**i**) optical and (**ii**) NIR-II images (c: post-resection). (**d**) Pre-laparotomy lymph node imaging and guided resection. (**e**) Ex vivo imaging of resected tissues. (**f**) Histopathology: (**i**,**ii**) tumor H&E; (**iii**) CK-7+ tumor cells; (**iv**,**v**) lymph node H&E. Reproduced with permission from Ref. [14]. Copyright 2023, ACS.

D-A-D structured NIR-II fluorophores employing benzothiadiazole (BBTD) electron-accepting moieties have garnered considerable research interest, offering enhanced tissue penetration (>5 mm depth), minimized photon scattering, and favorable biosafety profiles [146]. COF-980, engineered with BBTD chromophores, demonstrates exceptional photoresistance, restricted molecular diffusion (logP = 3.2 ± 0.1), and depth-tunable ROS generation (~8 mm), enabling effective photodynamic tumor ablation through FLI-guided PDT in 4T1 models [147]. Pu et al. [14] developed BBTD/DSPE-PEG nanoprobes through solvent displacement synthesis, achieving passive tumor accumulation for intraoperative guidance in simulated ovarian tumor microenvironments (Figure 8a). Systemic administration via caudal vein enabled detection of 2-mm subclinical lesions with complete autofluorescence suppression in the NIR-I spectral regime (Figure 8b,c). Pre-laparotomy NIR-II mapping revealed fluorescent lymph node basins (cervical/axillary/iliac; Figure 8d), with ex vivo specimens confirming target specificity in adnexal structures (Figure 8e). Histopathological correlation (Figure 8f) validated the platform’s diagnostic accuracy for micron-scale ovarian malignancies. Multifunctional NIR-II fluorophore development thus critically advances colorectal cancer theranostics and FLI innovation.

## 5. NIR-Based FMT Systems and Reconstruction Algorithms

FMT is a non-invasive, highly sensitive imaging modality that enables 3D tumor visualization through reconstruction of NIR fluorescent probe distributions [148]. FMT systems and reconstruction algorithms represent active research frontiers in biomedical imaging, particularly when integrated with NIR-II technology to enhance imaging depth and resolution [149]. In the past few decades, NIR-I fluorophores dominated as the primary spectral window for FMT (NIR-I FMT). Conventional reconstruction frameworks employ photon transport models and inverse solving algorithms optimized for NIR-I parameters. Nevertheless, this spectral range exhibits pronounced scattering, precluding precise in vivo tumor localization. In contrast, the NIR-II window demonstrates reduced photon scattering, enabling NIR-II FMT to achieve superior reconstruction fidelity and spatial co-registration accuracy [150].

To enhance FMT performance, multiple computational strategies have been investigated. Conventional tomographic approaches employ Monte Carlo (MC) simulations or reduced-order radiative transfer equation (RTE) models to characterize forward photon transport. Yugo et al. [151] validated FMI-guided PDT for 9–15 mm tumors using ICG-C11-enhanced MC simulations in prone-position breast phantoms. Nevertheless, MC methods remain computationally prohibitive, severely limiting clinical translation. Simplified RTE frameworks implement diffusion equations with Robin boundary conditions to approximate photon propagation. Li et al. [152] integrated locality-preserving projection (LPP) with sparse regularization to mitigate reconstruction ill-posedness, demonstrating ICG-based high-resolution imaging in heterogeneous phantoms and murine models. The fast iterative contraction threshold algorithm (R-FISTA) achieves 30% faster convergence through adaptive step-size optimization without sacrificing precision [153]. Cai’s group [154] developed a Gaussian-weighted neighborhood fusion lasso (GWNFL) method for NIR-I/II FMT reconstruction in hepatoma-bearing mice, reporting localization errors of 2.13 mm (NIR-I) versus 0.84 mm (NIR-II) and Dice coefficients of 0.22 versus 0.76, respectively, confirming NIR-II’s superior accuracy through reduced scattering.

Advances in machine learning have driven the implementation of neural network-based reconstruction strategies to resolve these limitations. Cao et al. [155] developed an excitation-gated fully connected network (EFCN) modeling NIR-II photon transport in biological tissues. In vivo studies in glioma-bearing murine models demonstrated EFCN-enhanced NIR-II fluorescence imaging performance, achieving a mean centroid error of 0.26 mm and Dice coefficient of 0.78. Darwan et al. [156] further validated that laser-scan excitation synergized with artificial neural networks enables reliable depth imaging (>10 mm) in thick tissue sections across wide fields of view.

Contemporary FMT systems and algorithms exhibit a paradigm shift from conventional iterative approaches to deep learning integration, resulting in marked enhancements in both reconstruction velocity and precision. Nevertheless, practical implementations necessitate resolving persistent challenges including noise attenuation, multimodal data amalgamation, and real-time processing efficacy. Future advancements should focus on interdisciplinary integration of cutting-edge technologies and refinement of deep learning architectures to optimize imaging reconstruction efficacy and fidelity for clinical diagnostic precision.

## 6. Conclusions and Prospect

NIR fluorescent materials demonstrate significant advantages in tumor imaging, including deep tissue penetration, high signal-to-noise ratios, and real-time monitoring capabilities, yet face persistent technical limitations. NIR-I cyanine dyes (e.g., MB, ICG) exhibit limited penetration depths, rendering them ideal for superficial malignancies such as cutaneous and breast cancers. While these agents achieve emission tails > 1000 nm in the NIR-II window for subcutaneous tumor visualization, their quantum yields remain suboptimal due to aggregation-caused quenching from intermolecular π-π stacking. Commercially available NIR probes (e.g., IR806) predominantly lack tumor-specific targeting ligands, relying on passive accumulation via the enhanced permeability and retention (EPR) effect, which induces nonspecific uptake and background interference. NIR-II nanomaterials, including nanoparticles, QDs, and rare-earth-doped materials, have a superior performance in penetration depth, signal-to-background ratio, and biocompatibility when compared to visible/NIR-I probes. However, QDs exhibit inherent heavy metal toxicity (Ag, Se, Pb), while rare-earth nanostructures show lower quantum yields compared to organic NIR-II dyes, compounded by aqueous solubility challenges and rapid photobleaching. Emerging organic NIR-II fluorophores, refined through surface engineering and core-shell architectures, demonstrate enhanced biocompatibility and safety profiles. Nevertheless, their clinical translation remains hindered by synthetic complexity and poor hydrophilicity. Furthermore, existing single-modality platforms fail to integrate synergistic therapeutic modalities such as PTT and PDT with real-time feedback.

To further improve the accuracy of NIR fluorescence image-guided surgery, several strategies can be adopted. The fluorescence wavelength and quantum yield can be regulated by extending the π-conjugated system or introducing electron-withdrawing/electron-donating groups to adjust the intramolecular torsion angle of the polymer. Develop activation-type nanoprobes to achieve the “OFF-to-ON” activation of NIR-II fluorescence. In addition, the persistent luminescence of nanoparticles is critical for advancing NIR bioimaging applications. The use of micelle and polymer coating can reduce the aggregation quenching effect and improve light stability at the same time. The penetration depth and signal-to-noise ratio are improved by designing a probe (NIR-III) that emits fluorescence within a longer wavelength range. The integration of multimodal synergistic therapy and imaging enables panoramic visualization from macroscopic anatomy to microscopic molecular level, thereby enhancing the therapeutic effect. For specific markers (such as pH, GSH, H_2_O_2_), intelligent probes that can provide real-time feedback on the metabolic status of tumors can be designed. Target-specific probes are crucial for the success of imaging applications. Developing NIR-II materials with multiple specific targets and stronger affinity may be a very promising research direction in the future. Future work should converge multidisciplinary technologies to engineer fluorescent probes with amplified signal intensity, synergized with advanced reconstruction algorithms for ultra-sensitive tumor imaging that addresses clinical precision medicine requirements.

## Figures and Tables

**Table 1:** Application of NIR fluorescent materials with different structures in imaging and treatment of tumors of different depths.

Type		Fluorophore	EX/EM Wavelength	Applications in Tumor	Object Element	Diagnosis/Treatment	References
Cyanine dye	ICG	5-Fu-ICG-MPEG-PCL	808/820 nm	Skin cancer	Nonspecificity	PTT synergistic chemotherapy	[20,21]
		ICG	808 nm	Oral squamous cell carcinoma (OSCC)	Podoplanin antibody	NIR-II FLI	[22]
		ICG	765/840 nm	Sentinel lymph node tumor	Nonspecificity	NIR-II FLI	[23]
	IRDye800CW category	IRDye800CW–E2	808/820 nm	Breast cancer	Estrogen receptor-α (ERα)	NIR-II FLI	[24]
		PPy @ CPT-HA-IRDye800CW	808 nm	Breast cancer	Nonspecificity	Chemotherapy, PTT, and PAI dual-modality imaging	[25]
		IRDye800CW	775/805 nm	Squamous cell carcinoma of the head and neck	Panizumab, cetuximab	NIR-II FLI	[26]
		IRDye800CW-NHS	808/1064 nm	HER^2+^ positive breast cancer	Trastuzumab	NIR-II FLI	[27]
	IR	IR1048	690/1064 nm	Liver cancer	Nonspecificity	PDT	[28]
		IR780	745/815 nm	Brain tumors	Nonspecificity	NIR-II FLI	[29]
Conjugated polymer	Donor-acceptor—donor (D-A-D)	FE-2PEG	808 nm	Colorectal cancer	Nonspecificity	NIR-II FLI	[15]
		IRFEP-FA-DOTA-Gd	808 nm	Liver cancer	Nonspecificity	NIR-II/PAI/MRI, FGS	[30]
		BTZ/Fe^2+^@BTF/ALD	1060 nm	bone tumor	Nonspecificity	PTT, chemotherapy, CDT	[31]
	Benzodithiadiazole (BBTD)	H4-PEG-PT	808/1060 nm	Bone tumour	Nonspecificity	NIR-II FLI	[32]
		αPD-L1@TPE-BT-BBTD	980 nm	Pancreatic cancer	Nonspecificity	NIR-II FI/PIT	[33]
Nano-particles (NPs)	Nanoparticle	Cu2-XSe	980/1064 nm	Liver cancer	Nonspecificity	NIR-II FLI, PTT/PDT combination therapy	[34]
		CAL@PG NPs	1064 nm	Liver cancer	Nonspecificity	NIR-II FLI, PTT/CDT/Chemotherapy	[35]
	Rare earth nanoparticles (REMP)	Nd@NaLuF4	808 nm	Breast cancer	Nonspecificity	NIR, MRI dual-modal imaging	[36]
		ErNPs@cRGD	808 nm	Breast cancer	Nonspecificity	NIR-II FLI	[37]
Quantum dot (QDs)	Ag_2_S	Ag_2_S@PEG-ABS	808 nm	Hypoxic tumor	Carbonic Anhydrase (CAIX)	Chemotherapy/PTT	[38]
		PNS/PEG-Ag_2_S QDs	808 nm	Liver cancer	Nonspecificity	NIR-II FLI, PTT	[8]
	PbS, CdS	PbS@CdS	980/1064 nm	Cervical cancer	Nonspecificity	NIR-II FLI	[39]

**Table 2:** Advantages and limitations of NIR-II fluorescent materials for imaging deep-seated tumors.

Types of NIR-II Fluorescent Materials	Advantages	Limitations	Ref.
Nanoparticle	Superior biocompatibility. High loading capacity of hydrophobic materials. High photothermal stability. Easy surface modification and specific targeting.	The synthesis process is complex. The biological safety awaits further study.	[89,90]
Quantum dots	The penetration depth can reach up to 15 mm. High photothermal stability. The emission wavelength is located in NIR-IIb (1500–1700 nm). High photothermal efficiency.	Typically contain heavy metals (e.g., Pb, Cd) and exhibit toxicity. Targeting efficiency is not high. Low solubility. The kidneys clear quickly.	[91,92,93]
NIR-II cyanine dye	High biocompatibility. Targeted modification is flexible. Excellent light stability with minimal photobleaching.	Most dyes emit wavelengths at the edge of NIR-I and require molecular engineering optimization to extend to NIR-IIb.	[79,94]
Rare earth nanomaterials	Good light stability. High signal-to-noise ratio. Stokes has a large displacement. Multimodal imaging.	Fluorescence quenching leads to a low quantum yield. A specific excitation light source is required. The synthesis process is complex.	[95,96,97]
Conjugated polymer	High photothermal efficiency. Structural adjustability. High light stability. Superior biocompatibility.	Low water solubility. Fluorescence quenching leads to a low quantum yield. Slow metabolism requires consideration of toxicity issues.	[98,99]
D-A-D small organic molecule	High photothermal efficiency. Rapid metabolism has good safety. Stokes has a large displacement. High light stability. Excellent compatibility.	Shorter emission wavelengths. Complex synthesis.	[100,101]

## Data Availability

Raw data and images are available.
