# Correction: Yu et al. Application of NIR Fluorescent Materials in Imaging and Treatment of Tumors of Different Depths. *Nanomaterials* 2025, *15*, 811

**DOI:** 10.3390/nano15191535

**Published:** 2025-10-09

**Authors:** Mengdi Yu, Xuan Liu, Shuqiong Wang, Ziyao Qin, Beibei Hu, Zhiwei Li, Shiguo Sun

**Affiliations:** 1 College of Chemical and Pharmaceutical Engineering, Hebei University of Science and Technology, 26 Yuxiang Road, Shijiazhuang 050018, China; 2023105164@stu.hebust.edu.cn (M.Y.); 2201060210@stu.hebust.edu.cn (X.L.); 2023105153@stu.hebust.edu.cn (Z.Q.); 2 Department of Applied Chemistry, Hengshui University, 1088 Heping West Road, Hengshui 053000, China; 15837046193@163.com; 3 Shanxi Key Laboratory of Natural Products & Chemical Biology, College of Chemistry & Pharmacy, Northwest A&F University, Xianyang 712100, China

In the original publication [1], the reference [94], “Dhole, N.V.; Dixit, V.V. Review of brain tumor detection from MRI images with hybrid approaches. *Multimed. Tools Appl.* **2022**, *81*, 10189–10220.”, has been retracted prior to this publication. The citation has now been replaced by “Wu, Y.; Hu, D.; Gao, D.; Liu, C.; Zheng, H.; Sheng, Z. Miniature NIR-II Nanoprobes for Active-Targeted Phototheranostics of Brain Tumors. *Adv. Healthc. Mater*. **2022**, *11*, 22023799”. The authors state that the scientific conclusions are unaffected. This correction was approved by the Academic Editor. The original publication has also been updated.
